# Thromboembolic hazard in hereditary hemorrhagic telangiectasia

**DOI:** 10.1002/jha2.471

**Published:** 2022-06-17

**Authors:** Pedro Asensi Cantó, Vicente Pablo Belloch Ripollés, M. Consejo Ortí Verdet, Pilar Lloret Madrid, Jürgen Solís Ruiz, Santiago Bonanad Boix

**Affiliations:** ^1^ Hematology Department Hospital La Fe Valencia Spain; ^2^ Radiology Department Hospital La Fe Valencia Spain

1

A 23‐year‐old woman with hereditary hemorrhagic telangiectasia was found in her bedroom presenting right‐side hemiparesis and aphasia. Brain computed tomography angiography (CTA) showed a complete occlusion of left‐medium cerebral artery (*Panel A*) that required mechanical thrombectomy.

CTA revealed the typical arteriovenous malformations in the lungs (*Panel B*) but not in the brain or liver. A bubble contrasted echocardiography ruled out intracardiacal shunts. Furthermore, repletion defects were found in subsegmentarial pulmonary arteries (*Panel C, amplified in the inset*). No thrombus was detected in lower extremity veins with Doppler‐ecography, and thrombophilia screening was negative.

Urgent thrombectomy, rehabilitation, and anticoagulation reduced neurological sequels to hand dizziness and mild concentration complaints. Arteriovenous malformations were embolized.

Hereditary hemorrhagic telangiectasia, also known as Rendu–Osler–Weber disease, is an autosomical‐dominant condition characterized by angiodysplastic lesions and arteriovenous malformations. Traditionally, the hemorrhagic manifestations have received all the attention. Nonetheless, arteriovenous malformations can bypass pulmonary circulation and permit paradoxical embolisms (see Figure 1).

**FIGURE 1:**
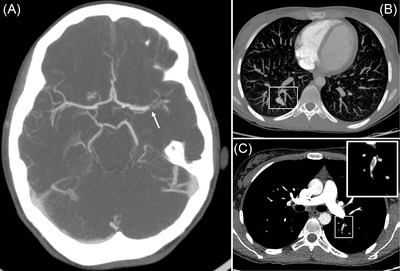
Panel A. Brain computed tomography angiography (CTA). Complete occlusion of left medium cerebral artery. Panel B. LungCTA. Typical arteriovenous malformations. Panel C. Lung CTA. Repletiondefects in subsegmentarial pulmonary arteries (*amplified in the inset*).

## CONFLICTS OF INTEREST

The authors declares they have no conflicts of interest.

Patient gave informed consent for the publication of this case report.

